# Evolution of COQ-Synthome Transcripts and CoQ Levels in Mice Tissues Along Aging: Effect of Resveratrol and Exercise

**DOI:** 10.3390/antiox14070800

**Published:** 2025-06-27

**Authors:** Catherine Meza-Torres, Iván Reyes-Torres, Tung Bui Thanh, Carmen Campos-Silva, Elisabet Rodriguez-Bies, Plácido Navas, Guillermo López-Lluch

**Affiliations:** 1 Department of Physiology, Anatomy and Cell Biology, Andalusian Centre for Developmental Biology, CABD-CSIC, CIBERER, Institute of Health Carlos III, Pablo de Olavide University, Carretera de Utrera km. 1, 41013 Sevilla, Spain; inmunolab02@uninorte.edu.co (C.M.-T.); tungasia82@yahoo.es (T.B.T.); ccampos@uloyola.es (C.C.-S.); erodbie1@upo.es (E.R.-B.); pnavas@upo.es (P.N.); 2 Grupo de Investigación en Inmunología y Biología Molecular, Universidad del Norte, Barranquilla 760046, Colombia; 3 OverT Bio, Inc., New York, NY 10016, USA; 4 Department of Pharmacology, University of Medicine and Pharmacy, Vietnam National University, Hanoi 10000, Vietnam; 5 Departamento de Ciencias de La Salud y Biomédicas, Facultad de Ciencias de la Salud, Universidad Loyola Andalucía, 41704 Seville, Spain; 6 Sports and Informatics Department, Pablo de Olavide University, 41013 Sevilla, Spain

**Keywords:** ubiquinone, coenzyme Q, aging, mice, mitochondria, antioxidant

## Abstract

The balanced control of the synthesis of CoQ along the life of the organism is essential to maintain the respiratory capacity at the mitochondria and the antioxidant protection of cell membranes and plasma lipoproteins. For this reason, we determined the levels of the transcripts of the CoQ-synthome along the life of mice in comparison with the levels of antioxidant enzymes and the levels of CoQ in these animals. Surprisingly, we found that some organs such as liver, kidney and heart show great differences in mRNA levels of some COQ-genes along life whereas others such as the brain or gastrocnemius muscle do not show differences. Interestingly, these differences were not related to the total amount of CoQ in these tissues, indicating a discrepancy between the transcript activity of the CoQ-synthome and the level of the product, CoQ. This likely responds to different regulatory levels including mRNA lifespan and CoQ turnover. Further, resveratrol and physical activity in old animals can modulate some transcripts but many of them are in an organ-dependent effect, indicating a different response to the regulators.

## 1. Introduction

Coenzyme Q (CoQ) is a molecule comprising a benzoquinone ring associated with an isoprenoid chain and is located in all cell membranes [1]. The main role of CoQ in mitochondrial metabolism is to transfer electrons from complexes I or II to III of the OXPHOS complex [2,3,4] but it also is involved in the catabolism of lipids by β-oxidation [5], synthesis of pyridine nucleotides [6], and one carbon metabolism and sulfide and amino acid metabolism [7]. In mitochondria, in the rest of cell membranes and in blood plasma lipoproteins, CoQ plays an essential antioxidant role preventing lipid peroxidation from being the principal endogenous lipophilic antioxidant in the organism [8,9]. This antioxidant activity depends on the membrane-associated antioxidant enzymes such as Cytochrome b_5_ reductase (CYTB_5_R), NAD(P)H quinone dehydrogenase 1 (NQO1) [10] and ferroptosis suppressor protein 1 (FSP1) [11] that transfer electrons from cytosolic NAD(P)H to the membrane-oxidized form of CoQ (ubiquinone) to reduce it to the reduced form (ubiquinol) [9]. Ubiquinol transfers electrons to membrane α-tocopherol or directly to oxidized lipids in a reaction in which ubiquinol is oxidized to ubiquinone. Moreover, recently it has been described that an oxidoreductase enzyme bound to the outer side of plasma membrane of hepatocytes is responsible for the maintenance of ubiquinol in blood plasma lipoproteins [12], preventing the oxidation of cholesterol.

**Figure 1:**
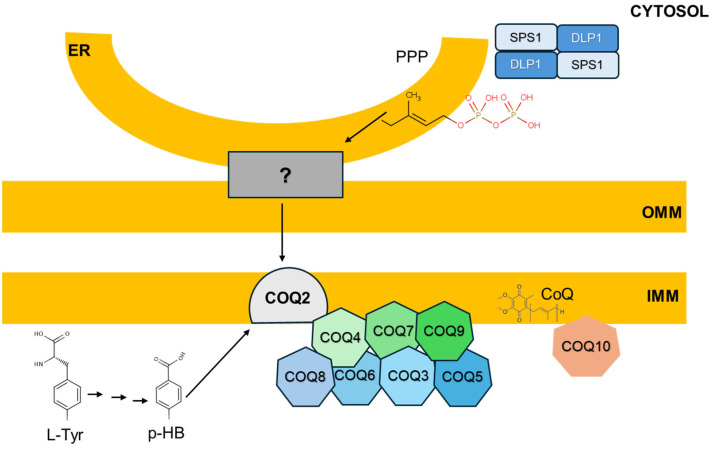
Representation of the process of synthesis of CoQ in mice. Members of the COQ1, SPS1 and DLP1 form a tetramer in cytosol to synthesize the precursor of the isoprenoid tail, polyprenyl-pyrophosphate (PPP). This is transported to the inner mitochondrial membrane (IMM) where COQ2 binds the tail to the precursor of the ring para-hydroxybenzoic acid (p-HB). After that, the other members of the synthome, from COQ3 to COQ9, serially modify the head to transform the molecule in the mature CoQ. COQ10 seems to contribute to the delivery of CoQ to active sites. The transport of the isoprenoid chain seems to depend on the presence of a complex that tethers the endoplasmic reticulum (ER) to the outer mitochondrial membrane (OMM).

CoQ is synthesized in mitochondria [13]. From mitochondria, CoQ is transferred to the rest of cell membranes by a mechanism not yet clarified but depending on the endomembrane secretory cycle [1]. The synthesis of CoQ is a complex process in which more than 13 different enzymes participate in, known as CoQ-synthome, mainly modifying the benzene ring [14] (Figure 1). These enzymes are codified by different genes located in different chromosomes in all the organisms and their regulation has not been completely clarified.

Aging is associated with a reduction in the synthesis of CoQ [15] and for this reason, many studies have been carried out to determine the effect of CoQ_10_ supplementation in humans against aging and age-related diseases [9,16,17,18]. However, to date, no information is available about the transcriptional regulation of the CoQ-synthome along aging and if this regulation affects CoQ synthesis. Post-transcriptional regulation plays an important role in the regulation of the lifespan of mRNA levels of the components of the CoQ-synthome [19] or protein levels [20,21].

Physical activity (PA) and the stilbene resveratrol (RSV) have demonstrated protective effects against oxidation and inflammation in different organs and tissues [22,23,24]. Further, both PA and RSV exert their effects in an age-dependent manner [24]. We have also found that RSV also changes mitochondrial physiology in mice under high-fat diet conditions [25,26] and RSV affects CoQ levels and activity of CoQ-dependent oxidoreductases in old animals in an organ-dependent effect [22,24,27].

Thus, in the present study, we wanted to know how the mRNAs of the members of the CoQ-synthome evolve along the lifespan of mice and if this is related with protein levels or CoQ levels. Further, we wanted to know the effect of PA or RSV on the CoQ-synthome transcriptome and if their effect is also organ-dependent in old animals.

## 2. Materials and Methods

### 2.1. Animals

This study was performed using male C57BL/6J mice, a common mice strain for laboratory purposes. Three age groups of at least 4 animals per group were studied: young (8 months), mature (18 months) and old (24 months). Animals were maintained in enriched environmental conditions in groups of 4–5 animals per polycarbonate cage. The colony was maintained under a 12 h light/dark cycle (12:00 AM–12:00 PM) with temperature (22 ± 3 °C) and humidity constant and controlled. Animals were maintained accordingly to a protocol approved by the Ethical Committee of the University Pablo de Olavide (CEEA 04/12) and following the international rules for animal research.

The group of old animals that were subjected to PA started the training procedure two months before the end of the experiment, at 22 months. Animals were trained following a plan consisting of one week of adaptation followed by a 5 days/week exercise on a treadmill (Treadmill Columbus 1055M-E50, Cibertec SA, Madrid, Spain) with 8% inclination for 20 min/day. The training protocol consisted of a 3 min warm-up at 5 m/min followed by an increase in the speed until reaching 20 m/min around the halfway point of the procedure and maintained until reaching 20 min of training.

In the case of the group treated with RSV, animals were randomly divided into two groups: control and RSV. Treatment started 6 months before the end of the experiment, at 18 months of age. The control group was provided with water containing 0,18% ethanol used as vehicle for RSV (180 µL ethanol/100 mL H_2_O) whereas in the group treated with RSV, the water contained RSV (180 µL of 0.1 mg/mL trans-RSV in ethanol/100 mL H_2_O) (Cayman Chemicals, Ann Arbor, MI, USA) that was provided in opaque bottles to avoid light-dependent decomposition of RSV. Previously we tested that mice drank around 4–5 mL/day and with a weight around 30 g, the calculated dose of RSV was around 500 μg/animal/day (16.5 mg/kg/day). RSV and fresh water were changed twice a week as was determined previously to avoid RSV decomposition [24].

At the end of the experiment animals were sacrificed by cervical dislocation in fasting conditions (O/N fasting). The liver, kidney, heart, brain and gastrocnemius muscle were immediately removed, frozen in liquid nitrogen and maintained at −80 °C until procedure and analysis.

### 2.2. CoQ-Synthome mRNA Levels Determination

The total mRNA from the different organs was obtained after homogenization with Trizol (Invitrogen, Life Technologies, Carlsbad, CA, USA) and extracted and cleaned with a RNeasy Mini kit (Qiagen Iberia S.L, Madrid, Spain). The possible remaining DNA was eliminated with RNAse free DNAse I (Sigma Aldrich, Barcelona, Spain). The quality and quantity of the mRNA was determined by a NanoDrop ND-1000 UV spectrophotometer (Thermo Scientific, Waltham, MA, USA). cDNA was obtained by using the iScript^TM^ cDNA synthesis system (Bio-Rad USA, Hercules, CA, USA).

The levels of mRNA of each gene were determined by quantitative qPCR using a CFX Connect Real-Time PCR Detection System (Bio-Rad, Hercules, CA, USA) using the iTaq Universal SYBR Green Supermix (Bio-Rad, Hercules, CA, USA). The oligonucleotides were designed through the tested primers published in PrimerBank (https://pga.mgh.harvard.edu/primerbank/ (accessed on 14 April 2020)) and produced by Eurofins MWG Synthesis GmnH (Ebersberg, Germany) (Table 1).

The best housekeeping gene used for reference was determined among four constitutive genes: β-actin, HSP90, HPRT and 18S by using the RefFinder (https://www.ciidirsinaloa.com.mx/RefFinder-master/ (accessed on 17 September 2021)) [28]. The β-actin mRNA was considered the most reliable housekeeping gene with the best score and was used for determinations.

### 2.3. Western Blot Analysis

The kidneys were homogenized with ice-cold tissue lysis buffer containing 2 mM Tris–HCl, 20 mM HEPES, 1 mM ethylenediaminetetraacetic acid, 70 mM sucrose, 220 mM manitol and 1 mM phenylmethylsulfonyl fluoride with protease inhibitors (Roche, Sant Cugat del Valles, Spain), and protein levels were determined by Bradford methods as previously described [24]

Protein homogenates were mixed with five times concentrated loading buffer containing sodium dodecylsulphate (SDS) and dithiothreitol (DTT) and were separated on a PAGE-SDS gel and transferred onto a nitrocellulose membrane. The total protein loading was determined by Ponceau S staining that was recorded for monitored transfer efficiency and quantification of total proteins for reference. Then, the membrane was blocked with 5% skim milk dissolved in 0.5 mM Tris–HCl (pH 7.5), 150 mM NaCl and 0.1% Tween-20 for 1 h at room temperature. Membranes were subsequently incubated with the corresponding primary antibody as indicated in Table 2. After three washes with Tris-buffered saline with 0.1% Tween-20 (TBST), blots were incubated with secondary horseradish peroxidase conjugated antibody in TBST with 5% skim milk at a 1:1000 dilution for 1 h at room temperature. Blots were then washed three times in TBST and developed using an enhanced chemiluminescence detection substrate Immobilon Western Chemiluminescent HRP Substrate (Merck Millipore, Madrid, Spain).

Protein levels were visualized by the ChemiDoc™ XRS+ System and compiled with Image Lab™ 4.0.1 Software (Bio-Rad Laboratories, Hercules, CA, USA). Immunodetected protein levels were corrected by whole protein loading determined by Ponceau Red staining.

### 2.4. CoQ Determination

The total CoQ were extracted and determined from brain, gastrocnemius muscle and liver homogenates as indicated previously [29]. One hundred pmol CoQ_6_ were used as internal control to check extraction efficiency. Sodium dodecyl sulfate (1%) was added to the sample and vortexed immediately during 1 min. Ethanol/isopropanol (95:5) in a proportion of 2:1 was added and mixed again during 1 min. For organic extraction, 600 μL hexane were added to the mixture and vortexed again for 1 min. After centrifugation at 1000× *g* for 10 min at 4 °C, the upper organic phase was removed and stored in a clean Eppendorf tube. Organic extraction was repeated twice more, and the three upper organic phases grouped. The CoQ-containing organic phase was dried by using a speed-vac at 35 °C. After that, dried lipid extract was dissolved in 60 μL ethanol and injected in HPLC system Beckman 166-126 (Beckman Coulter, Brea, CA, USA) by using a 20 μL loop. Lipid components were separated through a 15 cm Kromasil C-18 column (Sigma Aldrich, Barcelona, Spain) maintained at 40 °C in a continuous flux of 1 mL/min 65:35 methanol/2-propanol plus 1.42 mM lithium perchlorate mobile phase. The total levels of CoQ were detected by an electrochemical detector and expressed as pmol/mg protein.

### 2.5. Statistical Analysis

All the results are expressed as mean ± SD, at least n = 4 per group. Serial measurements were analyzed by using Student’s paired t-test and two-way ANOVA with Bonferroni’s post hoc test and normality test was determined by using the Shapiro–Wilk test using GraphPrism 8.0.2 program (GraphPad Software Inc., Solana Beach, CA, USA). Correlation of the mRNA levels of the genes was determined by a two-tailed Pearson’s correlation. Figures were drawn by using GraphPrism 8.0.2 program. The critical significance level α was 0.05 and then, statistical significance was defined as *p* < 0.05.

## 3. Results

### 3.1. An Organ-Dependent Regulation of the mRNA of the CoQ-Synthome Along Aging

**Figure 2:**
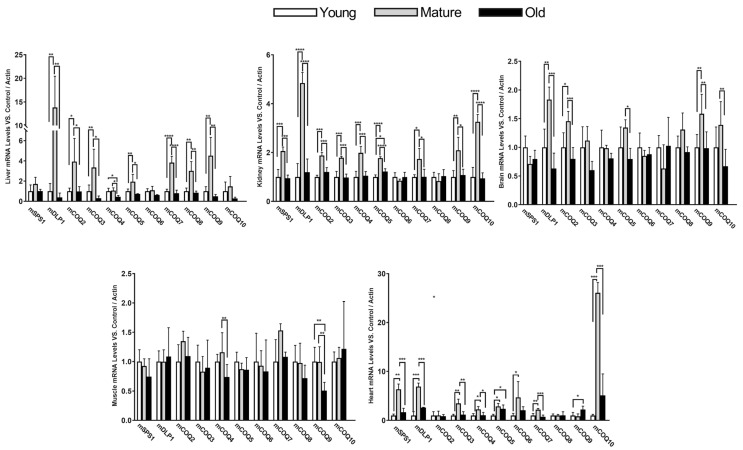
mRNA levels of COQ-genes in different organs along lifespan of mice. Data represent the mean ± SD of the levels of mRNA of the genes considering as reference the levels found in young animals and using as housekeeping gene, β-actin. Significative differences between the three age groups are indicated: * *p* < 0.05; ** *p* > 0.01; *** *p* < 0.001; **** *p* > 0.0001. n = 4 animals per age group.

We determined the levels of mRNA of the different members of the CoQ-synthome in different organs along mice life. We used young levels as a reference and β-actin as the best housekeeping gene indicated by RefFinder as indicated in Material and Methods (Figure 2). Interestingly, the liver and kidney showed very high differences in the levels of the mRNA of the different genes involved in CoQ synthesis along the life of mice. Mature organisms (18 months) showed higher levels of expression in most of the genes studied in both liver, kidney and heart. In the liver, these higher levels were found in mDLP1, mCOQ2, mCOQ3, mCOQ5, mCOQ7, mCOQ8 and mCOQ9. Interestingly, no changes in mCOQ4 mRNA levels were found in mature animals but a significant decrease in old animals were found in relationship with both young and mature animals. In the case of the kidney, all genes showed an increase in mRNA levels in mature animals vs. young and old animals except mCOQ6 and mCOQ8. And in the case of the heart, mCOQ2 and mCOQ8 did not show differences in expression and mCOQ9 showed a rise in old vs. young and mature animals whereas old animals maintained higher levels vs. young animals in the case of mCOQ5. Interestingly, in the heart, mCOQ10 showed a high rise in mature animals vs. all the other groups.

On the other hand, the brain and gastrocnemius muscle showed very low differences in mRNA levels along the lifespan of the animals, although in the brain, some genes showed low but significant increases in mature animals in comparison with both young and old animals. In the brain only five genes showed higher levels in mature animals vs. the other age groups: mDLP1, mCOQ2, mCOQ5, mCOQ9 and mCOQ10 and the levels of young and old animals were not different. In the case of the muscle, no increase was found in mature animals vs. young or old animals and only mCOQ4 and mCOQ9 showed a decrease in old animals vs. mature animals or young animals only in the case of mCOQ9.

Remarkably, in all the organs studied, the levels of expression in old animals (24 months) were very similar to those found in young animals (8 months). mDLP1 mRNA levels showed a rise in mature animals in comparison with young and old animals in the liver, kidney and brain but not in the gastrocnemius. This increase was very high in the liver and more moderate in the brain. Interestingly, the other component of the mCOQ1 complex, mSPS1, did not show such an increase in mature animals either in the liver or in the brain, indicating an imbalance between the expression of the two components of the mCOQ1-complex. Another interesting characteristic was that mCOQ6 did not show any remarkable variation in all the organs studied except in the heart, which showed a moderate increase in mature animals. Further, whereas in the liver, mCOQ10 did not show significant differences at different ages—although a tendency to decrease in old animals was found, in the kidney and in the heart—this gene did now show a clear increase in mature animals vs. the other age group.

In summary, all these determinations suggest that the members of the CoQ-synthome show different transcription regulatory mechanisms in different tissues and organs and at different ages.

**Figure 3:**
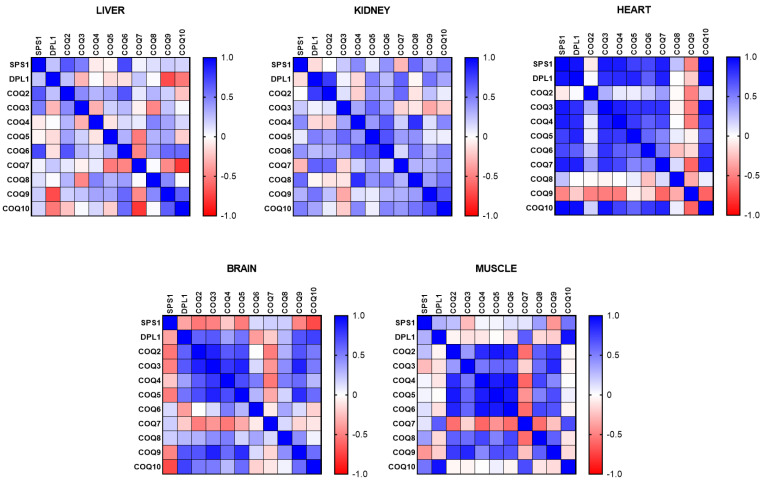
Correlation between the different COQ-genes in different organs along lifespan of mice. Data represent the level of positive correlation (blue) or negative correlation (red) among the different genes in the different organs. n = 12 per gene.

Taking into consideration these changes, we performed a correlation of the levels of mRNA of the different COQ genes to determine if the genes show any kind of coordinated expression in the different organs. In the liver, two small clusters were found; a cluster that comprised mSPS1, mDLP1 and mCOQ2, and another one with mCOQ9 and mCOQ10 that showed positive and significant correlation (Figure 3). On the other hand, mCOQ9 and mCOQ10 showed a negative correlation with mDLP1 and mCOQ7. Interestingly, in the kidney, that also showed high levels of expression in mature animals, this pattern of correlation was very different with many of the genes showing positive correlation between them being stronger between mDLP1 and mCOQ2, and mCOQ9 and mCOQ10, as was found in liver. (Figure 2). In the case of the heart, a big cluster of positive correlation was found between mSPS1, mDLP1, mCOQ3, mCOQ4, mCOQ5, mCOQ6 and mCOQ7. Interestingly mCOQ2 did not show correlation or even a negative one and mCOQ9 showed a strong negative correlation with many of the other genes. In the case of heart, mCOQ9 and mCOQ10 showed a strong negative correlation between them (Figure 2).

In the case of post-mitotic organs such as the brain and gastrocnemius muscle genes, mCOQ2 to mCOQ5 showed strong positive correlation between them in both organs. In the brain mSPS1 showed a strong negative correlation with many of the other genes. In the brain mCOQ9 and mCOQ10 showed positive correlation between them. (Figure 2). In the case of the gastrocnemius muscle, genes from mCOQ2 and mCOQ6 showed strong positive correlations between them in a response similar to that found in heart, but while in heart, mCOQ8 and mCOQ9 did not show correlation with the other genes; in the muscle they did show a strong and positive correlation. mCOQ7 was the gene that showed strong negative correlation with many of the other genes and also, as in the heart, mCOQ9 and mCOQ10 did not show correlation between them (Figure 2).

All these relationships demonstrate that the regulation of the expression of the genes of the CoQ-synthome and the control of the mRNA levels depends on the age of the animal and on the tissues or organs studied.

**Figure 4:**
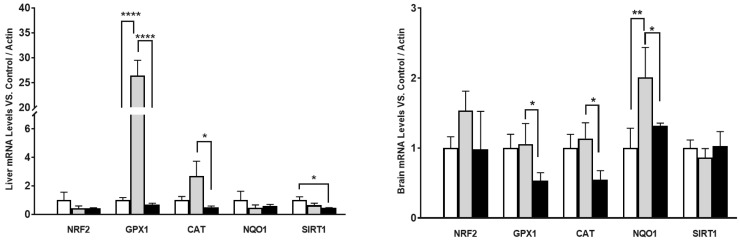
mRNA levels of antioxidant genes in different organs along lifespan of mice. Young animals: white columns, mature animals: grey colums, old animals: black columns. Data represents the mean ± SD of the levels of mRNA of the genes considering as reference the levels found in young animals and using, as housekeeping gene, β-actin. Significative differences between the three ages are indicated: * *p* < 0.05; ** *p* > 0.01; **** *p* > 0.0001. n = 4 animals per age group.

We also determined if some other genes involved in antioxidant protection presented a similar behavior in mRNA along aging. We determined the levels of different antioxidant genes and regulators such as Nuclear factor erythroid 2-related factor 2 (NRF2) and sirtuin 1 (SIRT1) in the liver and brain as representatives of the organs that showed higher differences and lower differences, respectively, in COQ-gene expression along lifespan (Figure 4).

Interestingly, the behavior was similar in some cases. In the liver, high differences in mRNA levels were found in H_2_O_2_-detoxifying genes such as glutathione peroxidase (GPX) and catalase (CAT) along the life of the mice. However, NRF2 and NQO1 showed a tendency to decrease with age, including mature animals and in the case of SIRT1, the decrease was significant between old and young animals. On the other hand, in the brain, the differences in expression were lower than in the liver, as with COQ-genes, and only NQO1 showed higher levels in mature animals in comparison with young animals. Glutathione peroxidase (GPX) and CAT did not show any increase in mature animals and did suffer a great decrease in old animals in comparison with both young and mature animals. No changes were found in NRF2 and SIRT1 genes. Thus, these results indicate that the different behavior in the expression of COQ-genes is also found in other genes and opens the possibility of a different regulation of gene expression in mature animals (Figure 4).

### 3.2. The Levels of mRNA of the CoQ-Synthome Components Do Not Correspond to the Protein Levels

**Figure 5:**
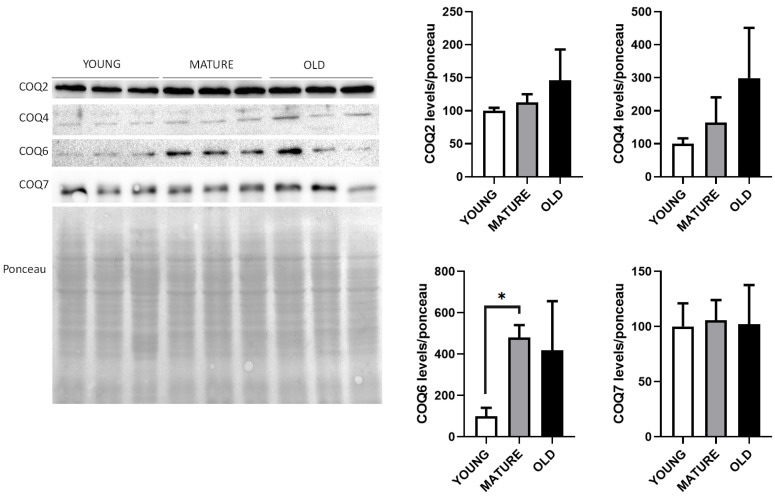
Liver CoQ-synthome protein levels along lifespan of mice. Representative WBs of Coq2, Coq4, Coq6 and Coq7 proteins in whole extract visualized by Red Ponceau staining. Quantification of WBs in reference to total loading is indicated as the mean ± SD considering as reference the levels found in young animals (n = 4 per group). Significative differences between the three ages are indicated: * *p* < 0.05.

We wanted to determine if the mRNA levels found in this study also corresponded to variations in the levels of their respective proteins. We used kidney extracts to determine the levels of Coq2, Coq4 and Coq7 proteins whose mRNA levels increased in mature animals and Coq6 whose mRNA levels did not change along lifespan (Figure 5).

Clearly, protein levels did not correspond to the levels of mRNAs since Coq2, Coq4 and Coq7 proteins did not increase in mature animals. In the case of the Coq4 protein, a trend to increase was found in mature and old animals. In the case of Coq6, that did not change at the mRNA level; a clear increase in mature animals and old animals was found significantly in the case of mature animals. In general, our determinations indicate that protein levels of these proteins do not correspond to the levels of mRNA.

### 3.3. Total CoQ Levels Are Not Related with the mRNA and Protein Levels of the CoQ-Synthome

**Figure 6:**
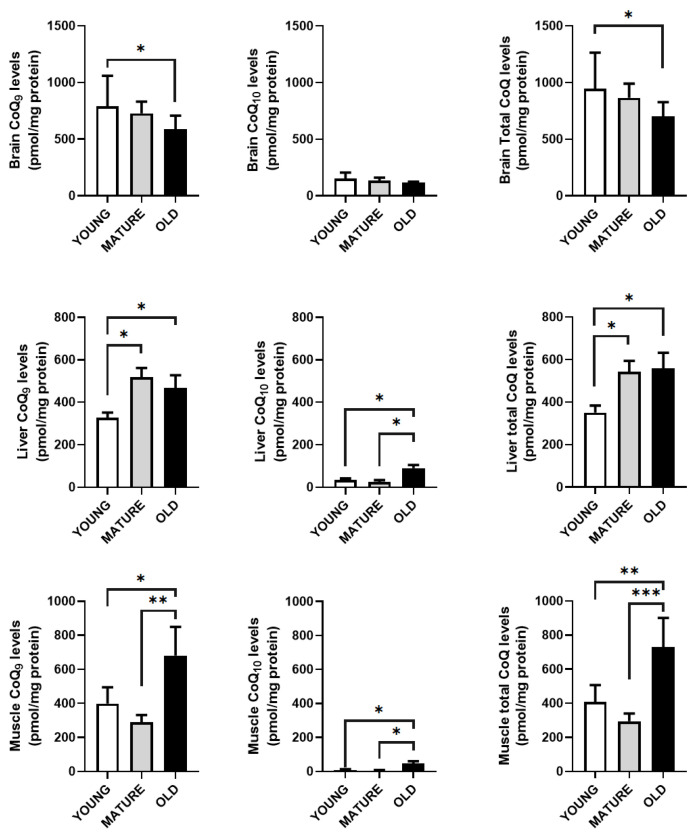
CoQ levels in brain, liver and gastrocnemius muscle along lifespan. Results indicate the mean ± SD of CoQ9 and CoQ10 in reference to mg of protein of total brain, liver and gastrocnemius muscle (n = 4). Significative differences between the three age groups are indicated: * *p* < 0.05; ** *p* < 0.01; *** *p* < 0.001.

We also measured the levels of CoQ_9_ and CoQ_10_, the two forms of CoQ found in mice, with CoQ_9_ bring the main form and the CoQ_10_ form making up around 10% of the total CoQ in the liver, brain and gastrocnemius muscle, to determine if the levels of mRNA and/or protein are indicative of the total level of CoQ in these tissues or organs (Figure 6).

Among the three organs studied, the brain showed the highest levels of CoQ per mg of protein. In young animals, the levels of CoQ_10_ were around 22% of the total levels in muscle, 16 % in brain and around 10% in liver. CoQ_9_ and total levels of CoQ in brain decreased in old animals. In the liver, a rise in the levels of CoQ_9_ and total CoQ levels were found in mature animals and maintained in old animals whereas in the case of CoQ_10_, the increase only was found in old animals. On the other hand, the gastrocnemius muscle only showed an increase in both CoQ_9_ and CoQ_10_ levels in old animals in comparison with young and mature animals.

In general, our results indicate that the levels of CoQ vary along the lifespan of the animals in a tissue and organ-dependent fashion and without any relationship with mRNA or protein levels of the members of the CoQ-synthome.

### 3.4. PA or RSV Modulate in Different Ways the Expression of COQ-Synthome Genes in Old Animals in an Organ-Specific Way

**Figure 7:**
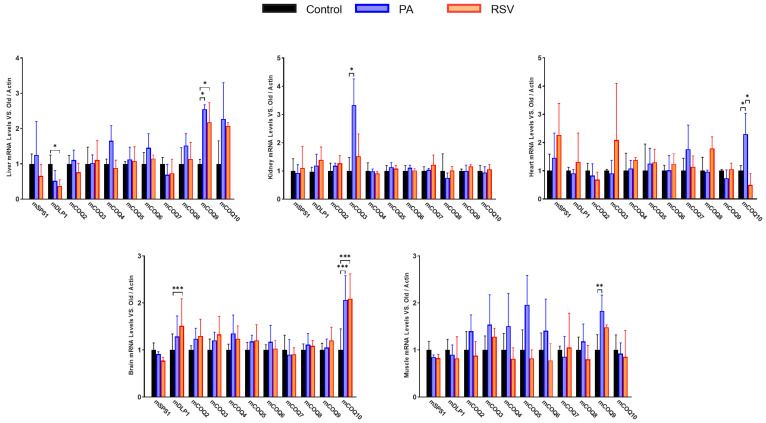
mRNA levels of COQ-genes in different organs in old mice that were trained (PA) or treated with RSV. Data represent the mean ± SD of the levels of mRNA of the genes considering, as reference, the levels found in young animals and using, as housekeeping gene, β-actin. Significative differences between the three ages are indicated: * *p* < 0.05; ** *p* > 0.01; *** *p* < 0.001. n = 4 animals per age group.

We also determined the effect of 2 months of PA or 6 months of RSV treatment in the expression of the different genes of the COQ-synthome in the different organs of old animals (Figure 7).

Interestingly, both PA and RSV affected in the same way the levels of mRNAs such as mCOQ10 in brain and liver. In the liver, mCOQ9 also responded in the same way to both stimuli. However, mDLP1 responded to RSV with a decrease in liver whereas in the brain, it increased. Interestingly, in the brain, mDLP1 increased after treatment with RSV whereas the mRNA of the other component of the mCOQ1 heterotetramer, mSPS1, did not show variation, indicating a different regulatory profile for both components.

In the case of the kidney, only mCOQ3 responded to PA whereas the rest of genes did not show any modification in their levels in comparison with the control. In the gastrocnemius muscle, PA showed a trend to increase the levels of many of the genes, although this increase was not significant except in the case of gene mCOQ9. Altogether, these results indicate that the response to PA or RSV also depends on the gene and the organ.

When the response of the different genes to PA or RSV was correlated, we found that PA induced a higher positive correlation of mRNA levels in the gastrocnemius muscle. In this tissue, mCOQ2, mCOQ3, mCOQ4, mCOQ5, mCOQ6, mCOQ8 and mCOQ9 showed a high correlation between them. On the other hand, mCOQ7 showed a high negative correlation with all of these genes. In the other organs, the correlation was very different depending on the organ, especially in the liver, which showed strong positive and negative correlations, whereas in the kidney, a general positive but modest correlation was found, and in the brain, mSPS1 showed moderate negative correlation with the other genes similarly to the response found with RSV.

**Figure 8:**
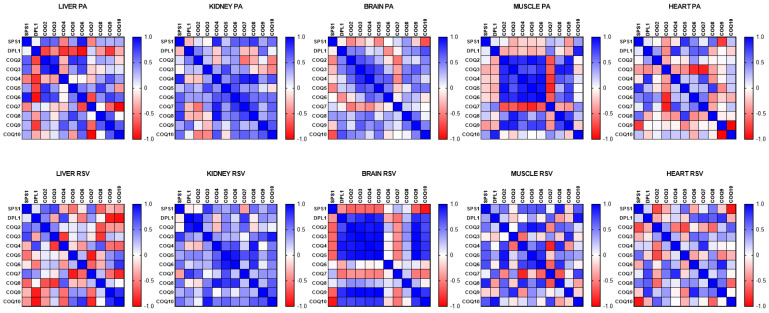
Correlation between the different COQ-genes in different organs along lifespan of mice. Data represent the degree of positive correlation (blue) or negative correlation (red) among the different genes in the different organs. n = 12 per gene.

On the other hand, RSV produced a higher coordination response in brain with mDLP1, mCOQ2, mCOQ3, mCOQ4, mCOQ5, mCOQ9 and mCOQ10, showing a high and positive correlation between them whereas mSPS1 and mCOQ7 showed strong negative correlations with the other mRNAs (Figure 8).

In the other organs only a few genes showed a similar high positive response to RSV. In the kidney many of the mRNA levels positively correlated between them but the correlation was mainly low or moderate. In the liver, mCOQ9 and mCOQ10 showed a high positive correlation and mDLP1, mCOQ2 and mCOQ3 showed a low positive correlation. The rest of the mRNAs showed a negative correlation with the other genes. In the gastrocnemius muscle, a variety of correlations was found between the genes with a higher correlation between mCOQ4, mCOQ5 and mCOQ6 as also was found in kidney.

All these responses indicate a complex profile of regulation affecting the genes of the CoQ-synthome and that the induction of them by PA or RSV depends on the organ studied.

### 3.5. PA or RSV Also Modulate in Different Ways the Expression of Antioxidant Genes in Old Animals in an Organ-Specific Way

**Figure 9:**
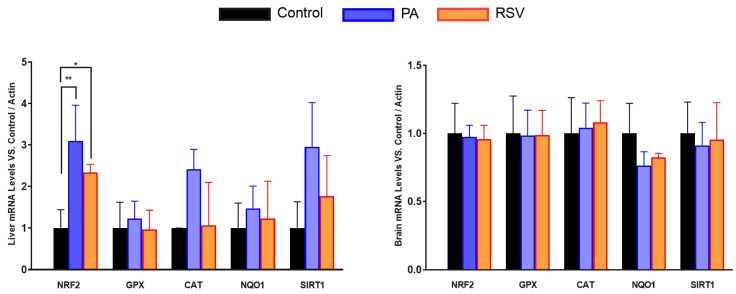
Levels of mRNA of different antioxidant genes in liver and brain after PA or RSV treatment. Data represent the mean ± SD of the levels of mRNA of the genes considering as reference the levels found in old animals and using actin as housekeeping gene. Significative differences between the three ages are indicated: * *p* < 0.05; ** *p* > 0.01. n = 4 animals per age group.

When we analyzed the levels of mRNA of antioxidant genes, we found that the effect of PA or RSV was higher in the liver than in the brain. In the liver, only NRF2 mRNA levels increased with both PA and RSV whereas a trend to increase was found in mRNA of CAT and SIRT1 with PA but not with RSV. In the case of the brain, no significant changes or trends were found in any of the mRNAs studied (Figure 9).

### 3.6. RSV or PA Increase Levels of CoQ in an Organ-Dependent Effect

**Figure 10:**
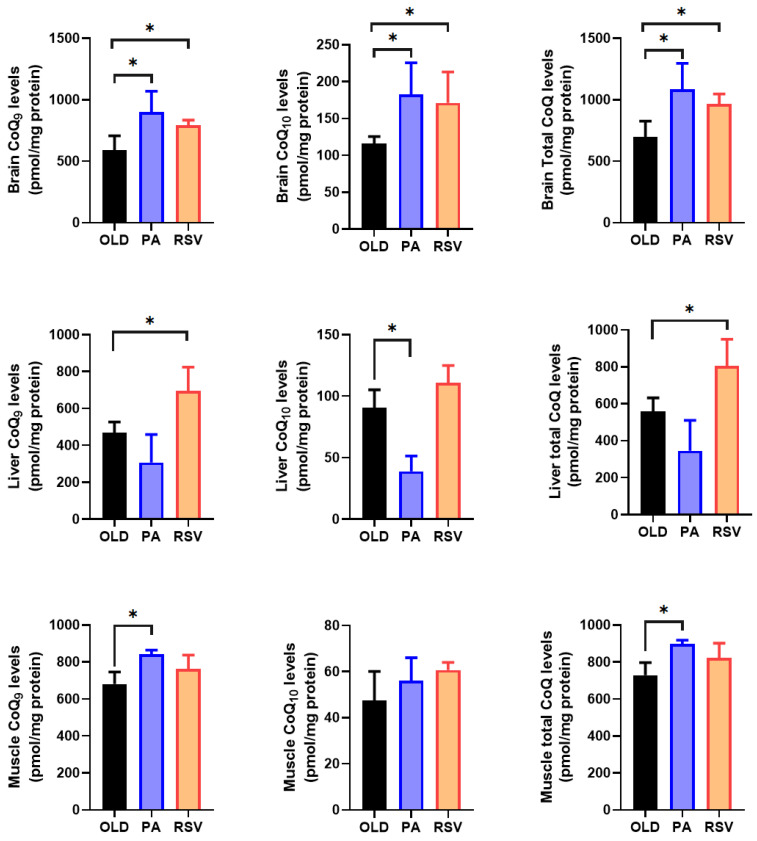
Levels of CoQ in brain, liver and gastrocnemius muscle after PA or RSV treatment. Data represent the mean ± SD of the levels of CoQ_9_, CoQ_10_ and total CoQ. Significative differences between the three ages are indicated: * *p* < 0.05. n = 4 animals per age group.

In the brain, both PA and RSV significantly increased CoQ_9_ and CoQ_10_ levels, reaching double the levels found in old animals (Figure 9). Interestingly, in this organ, both stimuli induced a significant increase in mCOQ10 mRNA levels (Figure 7). On the other hand, in the liver, although both PA and RSV increased the mRNA levels of both mCOQ9 and mCOQ10 (Figure 7), only RSV increased the levels of CoQ_9_ and CoQ_10_ and then, total CoQ (Figure 9), whereas PA slightly decreased these levels although not significantly. Finally, only PA significantly increased CoQ_9_ levels without affecting CoQ_10_ (Figure 9). In the gastrocnemius muscle, PA produced a tendency to increase the mRNA levels of many of the genes; this increase was significant with mCOQ9 (Figure 7). This induction of CoQ-synthome genes likely affected the rise in CoQ_9_ found in this tissue (Figure 10).

## 4. Discussion

Defects in CoQ biosynthesis cause CoQ deficiency syndrome [4]. Further, CoQ levels decline in some human and rodents tissues during aging [15,30] and, for this reason, we can consider this decrease as a secondary CoQ-deficiency [15] in which CoQ_10_ supplementation can be considered as an anti-aging agent therapy [16]. Studies agree on the fact that heart and lung CoQ levels decrease during aging, although in other cases the effect is different depending on the organism, including humans [15,30]. In agreement with our results, Beyer et al. (1985) showed that brain levels in mice do not show significant changes during the life of the animals, whereas liver levels increase [30]. However, our data show a rise in the levels of CoQ in the gastrocnemius muscle, whereas in the study of Beyer’s group a rise was found in mature animals decreasing to young levels in old animals [30]. This discrepancy can be due to different processes of the muscle and the differences in the procedures of isolation of the CoQ. In any case, a rise in CoQ in muscle can be explained by the decrease in white muscle fibers showing less mitochondria, the main location of CoQ in cells, during aging [31].

In any case, CoQ_10_ has shown therapeutic benefits in aging-related disorders, particularly in cardiovascular, metabolic disease and chronic kidney disease [32,33,34]. On the other hand, in some animal models with particular defects in CoQ synthesis, lower levels of CoQ are associated with a longer lifespan [35], although this effect is not found in all the organs [36]. This effect is likely due to the maintenance of enough CoQ to maintain physiological activity and the induction of mitohormesis [37].

Our results indicate that the expression of the genes involved in CoQ synthesis shows a complex regulatory profile in which some genes are regulated in a similar way depending on the age, the tissue or organ and the treatment. This regulatory landscape likely responds to common regulatory factors that remain to be discovered, and also the different location among the chromosomes of the different genes. The effect found in mice can also be extrapolated to human orans since the different genes involved in CoQ synthesis are also located in different chromosomes and only a few share chromosome and location. Even the mSPS1 and mDLP1 that form a heterotetramer with two proteins of Sps1 and two of Dlp1 to synthesize the isoprenoid chain of CoQ are found in chromosomes 2 and 10, respectively, and mCOQ8A and B that play a similar function, are located in chromosomes 1 and 7, respectively (Table 3). The same dispersion in the localization of genes of the CoQ_10_-synthome can be found in humans.

Further, even genes that share chromosome and location such as mPDSS1 and mCOQ4 do not respond in the same way to aging, PA or RSV.

Our results agree with other studies carried out in mutant mice affecting CoQ synthesis or mitochondrial physiology. For example, in two mouse models of COQ9 deficiency, *COQ9^Q95X^* and *COQ9^R239X^*, *COQ9^Q95X^* mice did not produce Coq9 protein, whereas *COQ9^R239X^* produced a truncated version of this protein [38]. As in our study, these two different mutations induced different changes in the expression of the CoQ-synthome genes and at the protein levels in an organ-dependent effect. In the cerebrum, the lack of the Coq9 protein did not affect COQ7, ADCK3/COQ8 or COQ5 but produced a small decrease in the COQ6 mRNA levels. This decrease was found in ADCK3/COQ8 in the kidney and in ADCK3/COQ8 and COQ5 in the muscle. In contrast, the truncated protein *COQ9^R239X^* did not affect the expression of any of the other CoQ-synthome genes. These results indicate that the lack of expression of one gene of the CoQ-synthome distinctively affects the expression of other genes but in an organ-dependent manner.

Further, in these *COQ9*-deficient models, no relationship between mRNA and protein levels were found. Whereas in the kidney, a lack of *COQ9* gene production was accompanied by a decrease in the ADCK3/COQ8 mRNA levels; the levels of the respective protein increased. Coq6 protein increased whereas Coq5 protein decreased, although their respective mRNA was not affected. Both the absence and the truncated form of *COQ9* enormously reduced the protein levels of Coq7 in the kidney and muscle. And in the muscle, a lack of *COQ9* and the truncated form also affected Adck3/Coq8, Coq5 and Coq6 protein levels, whereas only ADCK3/COQ8 and COQ5 were affected at the mRNA level [38].

Mitochondrial dysfunction is also associated with the regulation of the CoQ-synthome and the synthesis of CoQ. In five different models of OXPHOS dysfunction in the heart, both the transcriptome and proteome were strongly affected but the changes in protein levels did not reflect the changes at the transcriptional level [39]. In fact, among the 756 mitoproteins studied, nearly half of them changed significantly with age in control animals, whereas only less than 15% of the transcripts were affected.

In mutant animals, the levels of most of the proteins involved in CoQ synthesis were severely reduced, indicating that OXPHOS dysfunction drives CoQ deficiency. Similarly to the findings of our study, in OXPHOS-deficient mutants, many of the members of CoQ-synthesis were down-regulated but the mRNA levels of PDSS2, the ortologue of mDLP1) and ADCK3/COQ8 increased, indicating a different response of the genes to mitochondrial dysfunction [39].

All these results also agree with a previous study of our group in which high-fat diet reduced mRNA levels of all the CoQ-synthome genes, whereas neither protein levels nor CoQ levels were affected [40]. Furthermore, this same lack of relationship between mRNA levels and proteins has been found in human muscle, where some proteins do not respond to the increase or decrease in mRNA [41].

We cannot discard that the secondary deficiency of CoQ found in aging activates the induction of a survival mechanism that permits cells from adapting to the metabolic dysfunction associated with aging in a similar manner to the changes found in CoQ-primary deficiencies [42]. This would explain why the profile of old animals resembles the profile of young animals at the mRNA level.

In relationship with the levels of CoQ, some studies carried out in rats show a similar profile than in our study in which in mature animals there is a net increase in CoQ levels in comparison with young and old animals and a clear decrease is found, especially in heart [30]. Thus, the decrease in CoQ levels especially affect some organs, whereas others show an increase during aging.

Our results also indicate that PA and RSV can modulate the levels of mRNA of the different members of the CoQ-synthome in an effect that also depends on the organ. Interestingly, the main effect of PA and RSV affects mCOQ9 and mCOQ10 mRNA levels, two proteins that do not directly catalyze the modifications of the benzene ring but show regulatory and distributive functions. However, we cannot confirm that these increases are reflected by a rise in protein levels. In fact, physical exercise can lead to a rapid and robust increase in mRNA levels, although the debate about whether these changes end in modifications at the protein levels continues [43]. In humans under several sessions of high intensity interval training, during a 2-week intervention, some of the proteins involved in metabolism respond to the mRNA modifications whereas others increased despite the absence of any increase in the respective mRNA levels, such as PPARα and γ that regulate lipid metabolism. On the other hand, mitochondrial transcript factor mtTFA was increased by exercise without the corresponding increase in protein [44,45].

Furthermore, PA and RSV increase CoQ levels in brain whereas PA affected muscle and RSV liver levels of CoQ. These results indicate that each intervention significantly affects the main organ affected; the muscle in the case of PA and liver in the case of a bioactive compound such as RSV.

The use of both inducers could be important in the aging process. It has been suggested that mitochondria lose their ability to produce energy during maximal efforts during aging [46]; the increase of CoQ_9_, the main form of CoQ in rodents which is probably involved in OXPHOS activity, would improve this activity. This effect could help to improve walking performance [47] since mitochondrial function is strongly associated with muscle capacity. For this reason, it is likely that high levels of CoQ_10_ in plasma of humans are associated with higher PA capacity in old individuals [48]. On the other hand, the increase found in brain could explain why old people maintaining higher PA show higher cognitive capacity than sedentary people [49].

## 5. Conclusions

We can conclude that the levels of mRNA of the members of the CoQ-synthome show great differences along the life of the animals. They do not present a common regulation of expression along the life of the animals and that the mRNA levels vary depending on the age and stimuli in an organ-dependent response. The expression of these genes likely responds to different levels of regulation affecting mitochondrial turnover, mtUPR, regulation of mRNA lifespan and the lifespan of the CoQ product. Clearly, the level of mRNA of these genes does not represent the protein level in the tissue or organ or the levels of the product, CoQ.

Our results indicate that the behavior of the enzymes involved in the synthesis of CoQ and the CoQ levels cannot be extrapolated by the levels of mRNAs of the genes that codify these enzymes. A more complete study must be performed, including protein levels and CoQ levels.

## Figures and Tables

**Table 1:** Primers used in this study.

Gene	Acc Number	Forward (5′–3′)	Reverse (5′–3′)
*mSPS1*	NM_019501.4	5′-CATCAAAGGACACCAGCAATGT-3′	5′-GCACCACAATAATCGGTCTAAAGG-3′
*mDLP1*	NM_001168289.1	5′-ATGCTGACCTCCAGCCTTTT-3′	5′-GTCACACCTTTGCCAGCTTT-3′
*mCOQ2*	NM_027978.2	5′-GCCCACCAGCAGGACAAGAAAGAC-3′	5′-AGCCACAGCAGCGTAGTAGG-3′
*mCOQ3*	NM_172687.3	5′-GTGAGCCACCTGGAAATGTT-3′	5′-CCCACGTATGAGTGCCTTTT-3′
*mCOQ4*	NM_178693.5	5′-GGGGAGACCACAGGATGC-3′	5′-GTCGAGGGTAGACAGCGAGAT-3′
*mCOQ5*	NM_026504.3	5′-GGATTCCTTGGGAGGTTCA-3′	5′-GGGCAGTTCTTCAGCGTCT-3′
*mCOQ6*	NM_172582.3	5′-CGACGTGGTGGTGTCAGC-3′	5′-AGTTTCTCCAGGGCTTTCTTT-3′
*mCOQ7*	NM_009940.4	5′-TGATGGAAGAGGACCCTGAGAAG-3′	5′-GCCTGTATCGTGGTGTTCAAGC-3′
*mCOQ8/ADCK3*	NM_023341.3	5′-AGCAAGCCACACAAGCAGATG-3′	5′-CCAGACCTACAGCCAGACCTC-3′
*mCOQ9*	NM_026452.3	5′-CCCGAGTTTTCCCGTCC-3′	5′-TGGGCTCCTTCAGCAATG-3′
*mCOQ10*	NM_001039710.1	5′-TAAACAGAACCCTTCCACCG-3′	5′-CGAAATGCTGATAGTCCTCCA-3′
*SIRT1*	NM_019812.3	5’-TTGAAGATGCTGTGAAGTTACTG-3’	5’-GAAGGGTCTGGAGGGTCTG-3’
*GPX1*	NM_008160.6	5’- GAAGAACTTGGGCCATTTGG -3’	5’-TCTCGCCTGGCTCCTGTTT -3’
*CAT*	NM_009804.2	5’-TTATCCATAGCCAGAAGAG-3’	5’-CCAAAGAAAGAACAAGTCA-3’
*NRF2*	NM_010902.5	5’-CAGCATAGAGCAGGACAT-3’	5’-ACTATGATGGCGACAAAG-3’
*NQO1*	NM_008706.5	5’-CATTCAGAGAAGACATCATTCAACT-3’	5’-GCTTAGACTGGAGATACGATACT-3’
*SOD1*	NM_011434.2	5’-AATTACAGGATTAACTGAAGG-3’	5’-TAGGAGTGAGATTCTTTGTA-3’
β-actin	NM_007393.5	5′-TGACCGAGCGTGGCTACAG-3′	5′-GGGCAACATAGCACAGCTTCT-3′
mHSP90	NM_008302.3	5′-GTGCCTGGAGCTCTTCTCC-3′	5′-CGTCGGTTAGTGGAATCTTCAT-3′
mHPRT	NM_013556.2	5′-CAGTCAACGGGGGACATAAA-3′	5′-AGAGGTCCTTTTCACCAGCAA-3′
m18S	NR_003278.3	5′- TGACTCAACACGGGAAACCT-3′	5′-AACCAGACAAATCGCTCCAC-3′

**Table 2:** Primary antibodies used in this study.

Antibody	Host	Brand (Code)	Dilution
Anti-COQ2	Chicken	Agrisera (2005-165)	1:1000
Anti-COQ4	Rabbit	Proteintech (16654-1-AP)	1:1000
Anti-COQ6	Rabbit	Proteintech (12481-1-AP)	1:1000
Anti-COQ7	Rabbit	Proteintech (15083-1-A)	1:1000
Anti-Chicken HRP	Rabbit	Merck (12-341)	1:10,000
Anti-Rabbit HRP	Goat	Thermo Fisher (31460)	1:3000

**Table 3:** Mouse genes involved in CoQ synthesis with the chromosome and the location (https://www.ncbi.nlm.nih.gov/gene (accessed on 20 April 2025)).

Gene	Name	Gene ID	Chromosome Location	Protein
***mSPS1***	Prenyl(Solanesyl)diphosphate syntase subunit 1	56075	Chr2-NC_000068.8	Q9CZQ1
***mDLP1***	Prenyl(Solanesyl)diphosphate syntase subunit 1	71365	Chr10-NC_000076.7	Q33DR3
***mCOQ2***	4-hydroxybenzoate polyprenyltransferase	71883	Chr5-NC_000071.7	Q66JT7
***mCOQ3***	Ubiquinone biosynthesis O-methyltransferase	230027	Chr4-NC_000070.7	Q8BMS4
***mCOQ4***	Ubiquinone biosynthesis protein COQ4	227683	Chr2-NC_000068.8	Q8BGB8
***mCOQ5***	2-methoxy-6-polyprenyl-1,4-benzoquinol methylase	52064	Chr5-NC_000071.7	Q9CXI0
***mCOQ6***	Ubiquinone biosynthesis monooxygenase COQ6	217727	Chr12-NC_000078.7	Q8R1S0
***mCOQ7***	5-demethoxyubiquinone hydroxylase	12850	Chr7-NC_000073.7	P97478
***mCOQ8A/ADCK3***	Atypical kinase COQ8A	67426	Chr1-NC_000067.7	Q60936
***mCOQ8B/ADCK4***	Atypical kinase COQ8B	76889	Chr7-NC_000073.7	Q566J8
***mCOQ9***	Ubiquinone biosynthesis protein COQ9	67914	Chr8-NC_000074.7	Q8K1Z0
***mCOQ10A***	Coenzyme Q-binding protein COQ10 homolog A	210582	Chr10-NC_000076.7	Q8BV28
***mCOQ10B***	Coenzyme Q-binding protein COQ10 homolog B	80219	Chr1-NC_000067.7	Q3THF9
***mADCK2***	AarF domain containing kinase 2	57869	Chr6-NC_000072.7	Q6NSR3

## Data Availability

All the data presented are contained within the article.

## Abbreviations

The following abbreviations are used in this manuscript:

CAT	Catalase
CoQ	Coenzyme Q
COQ	Gene of the CoQ-synthome
CYTB5R	Cytochrome b5 reductase
FSP1	Ferroptosis suppressor protein 1
GPX	Glutathione peroxidase
NQO1	NAD(P)H quinone dehydrogenase
OXPHOS	Oxidative phosphorylation
NRF2	Nuclear factor erythroid 2-related protein 2
PA	Physical activity
RSV	Resveratrol
